# Formal recognition of the species of *Oreosaurus* (Reptilia, Squamata, Gymnophthalmidae) from the Sierra Nevada de Santa Marta, Colombia

**DOI:** 10.3897/zookeys.691.13595

**Published:** 2017-08-17

**Authors:** Santiago J. Sánchez-Pacheco, Pedro M. Sales Nunes, Sergio Marques-Souza, Miguel T. Rodrigues, Robert W. Murphy

**Affiliations:** 1 Department of Ecology and Evolutionary Biology, University of Toronto, 25 Willcocks Street, Toronto, ON M5S 3B2, Canada; 2 Department of Natural History, Royal Ontario Museum, 100 Queen’s Park, Toronto, ON M5S 2C6, Canada; 3 Departamento de Zoologia, Instituto de Biociências, Universidade de São Paulo, São Paulo, CEP 05508-090, SP, Brazil; 4 Departamento de Zoologia, Centro de Biociências, Universidade Federal de Pernambuco, Av. Professor Moraes Rego S/n, Cidade Universitária 50670-901, Recife, PE, Brazil

**Keywords:** Microteiid lizard, *Oreosaurus serranus*, nomenclatural recognition, *Proctoporus serranus*, *Proctoporus specularis*, *nomina nuda*, South America, taxonomy

## Abstract

*Oreosaurus* is one of the two genera extracted from the former *Riama* *sensu lato*, which was recently recognized as polyphyletic. *Oreosaurus* is a small clade (five named and two undescribed species) of montane gymnophthalmid lizards and exhibits an exceptional distributional pattern. Its nominal and undescribed species are discontinuously distributed on the Cordillera de la Costa of Venezuela, the tepuis from the Chimantá massif in Venezuela, the highlands of the island of Trinidad, and the Sierra Nevada de Santa Marta in Colombia (SNSM). Herein, we describe the species of *Oreosaurus* that is endemic to the SNSM. Historically, this species associates with two names that are currently *nomina nuda*: *Proctoporus serranus* and *P. specularis*. Formal nomenclatural recognition of *Oreosaurus serranus* **sp. n.** renders *specularis* a permanently unavailable name for this taxon. *Oreosaurus serranus* **sp. n.** is the sister of all remaining congeners, and differs primarily from them in having only one pair of genial scales, as well as a unique pattern of scutellation. We provide an identification key to the species of *Oreosaurus*.

## Abstract (es)

**Resumen**

*Oreosaurus* es uno de los dos géneros que fueron extraídos de *Riama* *sensu lato*, el cual fue reconocido recientemente como polifilético. *Oreosaurus* es un clado pequeño (cinco especies nominales y dos indescritas) de gimnoftálmidos de montaña y presenta un patrón de distribución excepcional. Sus especies nominales e indescritas se distribuyen discontinuamente sobre la Cordillera de la Costa de Venezuela, los tepuyes del macizo de Chimantá en Venezuela, las tierras altas de la isla de Trinidad, y la Sierra Nevada de Santa Marta en Colombia (SNSM). Describimos la especie de *Oreosaurus* que es endémica de la SNSM. Históricamente, esta especie se asocia con dos nombres que son *nomina nuda*: *Proctoporus serranus* y *P. specularis*. El reconocimiento formal de *Oreosaurus serranus* **sp. n.** hace que *specularis* sea un nombre permanentemente indisponible para este taxón. *Oreosaurus serranus* **sp. n.** es la especie hermana de todos los congéneres restantes, y se diferencia de ellos principalmente por tener un único par de escamas geneiales, así como por presentar un patrón único de escamación. Proveemos una clave de identificación para las especies de *Oreosaurus*.

## Introduction

*Oreosaurus* Peters, 1862 (Reptilia: Gymnophthalmidae) contains five named species of montane lizards that have discontinuous distributions on the Cordillera de la Costa and tepuis from the Chimantá massif in Venezuela, and the Aripo northern range in the Caribbean island of Trinidad (Sánchez-Pacheco et al. 2017). An additional species that is the sister of all remaining congeners and is endemic to the Sierra Nevada de Santa Marta in Colombia (SNSM) remains undescribed. Sánchez-Pacheco et al. (2017) referred to it as “Sierra Nevada”.

Over 30 years ago, Ayala and Castro reviewed the Colombian lizard fauna in their unpublished but widely distributed book “Lizards of Colombia”. Their work included brief descriptions of several species and they referred to informal specific epithets associated with authors to indicate that formal descriptions were not yet published, but were forthcoming. Among these species, Ayala and Castro included “*Proctoporus*” “*serranus*”, a gymnophthalmid lizard from the Serranía de San Lorenzo, SNSM, and they provided a reference for the description (Harris, dated to 1984). However, Harris' formal description of this taxon was never published. Although Ayala and Castro included a brief description (based on an undetermined number of specimens), the name “*serranus*” is a *nomen nudum* because it does not have a reference, and therefore fails to conform to ICZN (1999) Art. 11. Similarly, Ayala (1986) published a list of Colombian lizards, which included undescribed species referred to names within quotes (“”) and associated with authors to indicate imminent formal descriptions. Most of these names were the same ones provided by Ayala and Castro (unpublished data), the exception being “*Proctoporus*” “*specularis*”, also from San Lorenzo, SNSM. Nevertheless, both the locality and the given reference (Harris, but this time dated to 1986—also never published) were strongly suggestive that “*serranus*” and “*specularis*” referred to the same species. However, in accordance with ICZN (1999) Art. 13, the absence of a description for “*specularis*” (Ayala 1986) renders this name a *nomen nudum*.

While carrying out field work in the SNSM, we had the opportunity to collect a series of specimens that conform to the unpublished description of “*serranus*”. Two terminals labeled “Sierra Nevada” 1 and 2 were included in a recently published phylogenetic analysis of *Riama* Gray, 1858 *sensu lato* (Sánchez-Pacheco et al. 2017), which recovered this species as part of the resurrected *Oreosaurus*. Although “*serranus*” and “*specularis*” are currently *nomina nuda*, and by definition unavailable names (i.e., they fail to conform to ICZN Arts. 11 and 13), both of them have reached the modern literature (Rueda-Almonacid et al. 2012 and de Albuquerque et al. 2012, respectively). A *nomen nudum* can be made available (or validated) if it is published again in a way that meets the criteria of availability (ICZN 1999). *Anadia altaserrania* Harris & Ayala, 1987, another endemic gymnophthalmid lizard from the SNSM, is a pertinent example. It was included in Ayala and Castro’s unpublished book (with reference to Harris, Ayala and Castro, 1984) and listed by Ayala (1986; this time with reference to Harris and Ayala, 1986), but finally published formally by Harris and Ayala (1987). The situation with *Oreosaurus* is not unlike that of *Anolis* in which Poe et al. (2009) provided examples of *nomen nudum* species of *Anolis* lizards listed by Ayala (1986). Below we provide a name and a description for the species of *Oreosaurus* from the SNSM.

## Materials and methods

For comparative purposes, specimens of *Oreosaurus achlyens* (Uzzell, 1958), *O. luctuosus* Peters, 1862, *O. shrevei* (Parker, 1935) and the undescribed *O.* “Venezuela” were examined (Appendix 1). Data for *O. mcdiarmidi* (Kok & Rivas, 2011) and *O. rhodogaster* (Rivas et al., 2005) were taken from the literature (Kok and Rivas 2011 and Rivas et al. 2005, respectively). Measurements (snout-vent length [SVL] and tail length) were taken to 0.1 mm with a digital caliper. Sex was determined by noting the presence of hemipenes in males and/or secondary sex characters, such as the number of femoral pores. To facilitate comparisons with other species of *Oreosaurus*, scutellation and head-scale terminology follows Kizirian (1996). Bilateral variation is reported as left/right. Hemipenes were prepared following the procedures described by Manzani and Abe (1988) as modified by Pesantes (1994) and Zaher (1999). The retractor muscle was severed manually and an everted organ was filled with stained petroleum jelly. Following Uzzell (1973) and Nunes et al. (2012), calcareous hemipenial structures were stained in an alcoholic solution of alizarin red. Terminology follows Dowling and Savage (1960), Savage (1997) and Nunes et al. (2012).

The following collection abbreviations are used herein: AMNH (American Museum of Natural History, New York), EBRG (Museo de la Estación Biológica de Rancho Grande, Maracay, Venezuela), MCZ (Museum of Comparative Zoology, Harvard University, Cambridge, USA), ROM (Royal Ontario Museum, Toronto, Canada), and USNM (National Museum of Natural History, Washington D.C., USA).

## Species description

### Oreosaurus serranus sp. n.

Taxon classificationAnimaliaSquamataGymnophthalmidae

http://zoobank.org/5BB0FB0B-47E8-4788-BD79-2784FF91F63F

Figures 1 , 2 , 3

#### Holotype.

**Figure 1.**
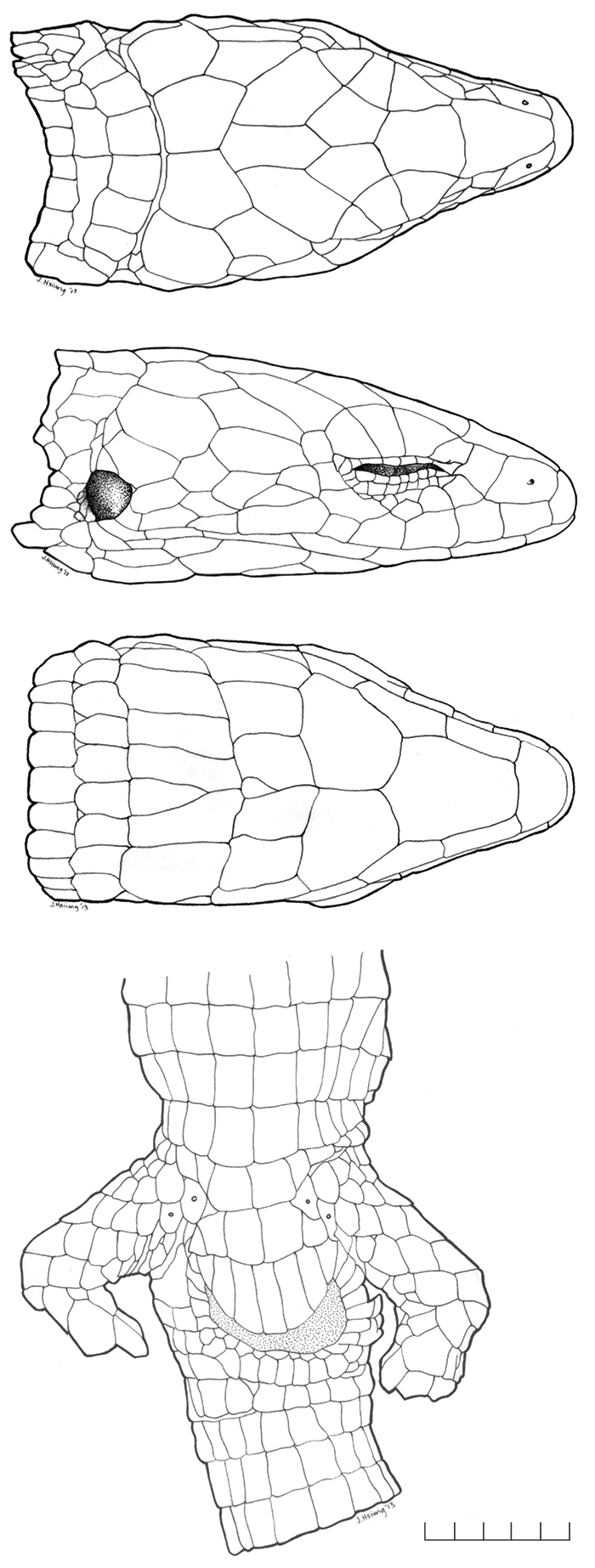
*Oreosaurus serranus* sp. n. (holotype, ROM 53608 [70.4 mm SVL]). Dorsal, lateral and ventral views of the head, and ventral view of the pelvic region.

ROM 53608 (field number JJS 548; Fig. 1), an adult female collected by S.J.S-P., P.M.S.N., S.M.S, Liliana Saboyá-Acosta, Jhon Jairo Ospina-Sarria, Sandy B. Arroyo, and Mariane Targino Rocha in Colombia, Sierra Nevada de Santa Marta, Departamento de Magdalena, headwaters of the Río Guachacos, Corregimiento de Minca, finca Vista Hermosa, approximately 2156 m, June 2013. This locality is situated at approximately 11°05'N, 74°01'W.

#### Paratypes.

**Figure 2.**
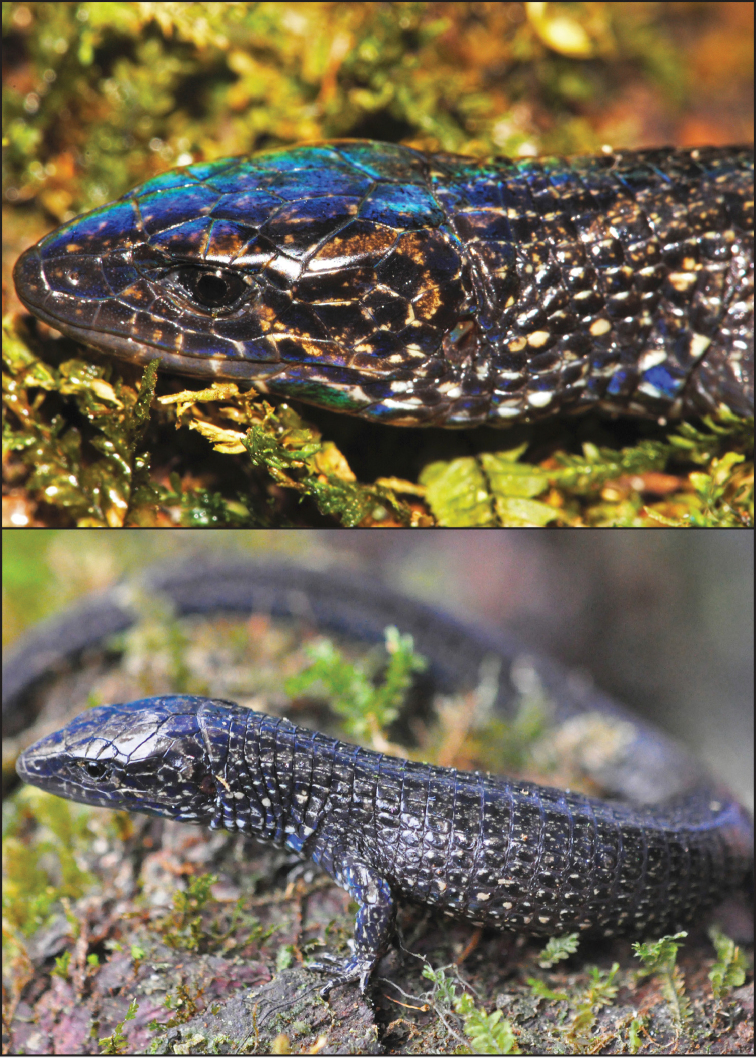
*Oreosaurus serranus* sp. n. (paratype, ROM 53609 [68.6 mm SVL]) in life. Photos: S.M.S (top) and Jhon Jairo Ospina-Sarria (bottom).

ROM 53609 (adult female, Fig. 2), ROM 53610 (subadult male), ROM 53611 (subadult female), ROM 53612–13 (juvenile females), and ROM 53614 (juvenile male), all with same data as holotype.

#### Diagnosis.

*Oreosaurus serranus* sp. n. can be distinguished from all its congeners by the number of genial pairs (1 in *O. serranus* sp. n. versus 2 in the other species). It also differs from all other species of *Oreosaurus*, except *O. mcdiarmidi*, by the number of supraoculars (3 in *O. serranus* sp. n. and *O. mcdiarmidi* versus 4 in the other species), and dorsal scale relief (smooth in *O. serranus* sp. n. and *O. mcdiarmidi* versus keeled or slightly keeled in the other species). *Oreosaurus serranus* sp. n. also differs from *O. mcdiarmidi* by the absence of prefrontal scales (present in *O. mcdiarmidi*).

#### Description.

*Oreosaurus serranus* sp. n. possesses the following characteristics: (1) maximum known SVL in males 60 mm (*n* = 2), in females 70.4 mm (*n* = 5); (2) frontonasal equal to or longer than frontal; (3) prefrontal scales absent; (4) nasoloreal suture complete [= loreal present]; (5) supraoculars three, all in contact with ciliaries; (6) superciliary series incomplete, formed only by the anteriormost superciliary scale; (7) supralabial-subocular fusion absent; (8) postoculars two; (9) postparietals two; (10) supratympanic temporals two; (11) genials in one pair; (12) dorsal scales rectangular, juxtaposed, smooth; (13) nuchal scales smooth; (14) longitudinal dorsal scale rows 10–11; (15) transverse dorsal scale rows 33–36; (16) ventral scales smooth, in 21–22 transverse scale rows; (17) lateral scale rows (oval, non-granular scales) 4–6; (18) femoral pores per hind limb in males 7–9, in females 2–3 (located proximally); (19) scales between medialmost femoral pores two; (20) subdigital scales on toe I four; (21) anterior cloacal plate scales four or six; (22) posterior cloacal plate scales seven; (23) dorsum dark brown to black with fine brown mottling; distinct dorsolateral stripes absent; lateral ocelli (i.e., white spots surrounded by dark blotches) absent (white or cream spots instead); venter black with conspicuous whitish spots mostly on scale sutures; (24) hemipenial body globose, slightly bilobed, ornamented by 14–15 chevron-shaped flounces on each side.

#### Description of holotype.

Adult female (Fig. 1), SVL = 70.4 mm, tail length = 72.4 mm; head scales smooth, glossy; rostral scale wider than long, higher than adjacent supralabials, in contact with frontonasal, nasals, and anteriormost supralabials posteriorly; frontonasal roughly quadrangular, longer than wide, widest posteriorly, equal in length to frontal, in contact with nasals and loreals laterally, and frontal posteriorly; prefrontals absent; frontal longer than wide, anterior suture convex, lateral sutures concave, posterior suture angular with point directed posteriorly, in contact with anteriormost supraoculars and superciliaries posterolaterally, and frontoparietals posteriorly; frontoparietals pentagonal, in contact anterolaterally with all supraoculars on the left side and second and third supraoculars on the right side, and posteriorly with parietals and interparietal; interparietal hexagonal, longer than wide, lateral sutures concave, in contact with parietals laterally, postparietals posteriorly; parietals in contact with third supraoculars anterolaterally, dorsalmost temporal and postocular scales laterally, and postparietals posteriorly; postparietals pentagonal, two, in broad contact; supraoculars three, all in contact with ciliaries. Nasoloreal suture complete, nasal quadrangular; loreal quadrangular, not in contact with second supralabial; superciliary series incomplete, formed only by the anteriormost superciliary scale, which barely extends onto dorsal surface of head, and lies between loreal, frontal, first supraocular, and anteriormost ciliaries; palpebral disc of lower eyelid divided into three large, unpigmented scales; frenocular quadrangular, in contact with loreal and nasal anteriorly; circumorbital scales between posteriormost supraocular and frenocular five; postoculars two; temporals smooth, glossy, polygonal; supratympanic temporals two; supralabials seven; infralabials four. Mental wider than long, in contact with anteriormost infralabials and postmental posteriorly; postmental roughly pentagonal, posterior suture angular with point directed posteriorly, in contact with first and second infralabials laterally; genials in one pair, roughly quadrangular, in contact with second and third infralabials; scale rows between genials and collar fold (along midventral line) eight, medialmost scales of posteriormost scale row distinctly enlarged, smooth; posteriormost gular row enfolded posteriorly, concealing one small scale row; lateral neck rounded, smooth.

Dorsal scales rectangular, longer than wide, juxtaposed, smooth, in 35 transverse rows; longitudinal dorsal scale rows at fifth transverse ventral scale row nine, at 10^th^ transverse ventral scale row 10, at 15^th^ transverse ventral scale row 11; lateral scale rows at fifth transverse ventral scale row 6/5, at 10^th^ transverse ventral scale row four, at 15^th^ transverse ventral scale row four; lateral scales on body near insertion of forelimb small to granular; ventral scales quadrangular, smooth; complete transverse ventral scale rows 22; longitudinal ventral scale rows at midbody 10; anterior cloacal plate scales six; posterior cloacal plate scales seven, medialmost scale with a horizontal suture; scales on tail rectangular and juxtaposed; midventral subcaudals smooth, wider than adjacent scales, nearly square. Femoral pores per hind limb two, located proximally; scales between medialmost femoral pores two.

#### Coloration of holotype.

In life, dorsal ground color dark brown to black with fine brown mottling; dorsal surfaces of head, body and tail with an iridescent bluish shine. White or cream spots laterally from neck to posterior portion of body, becoming less distinct posteriorly. Ventral surfaces of head and body predominantly black, with conspicuous whitish spots mostly on scale sutures; subcaudally black without spots. In preservative (70% ethanol), dorsal ground color brown with fine light brown mottling; dorsal surfaces of head, body and tail without the iridescent bluish shine. Ventral surfaces of head and body brown with cream spots on scale sutures.

#### Hemipenial morphology.

**Figure 3.**
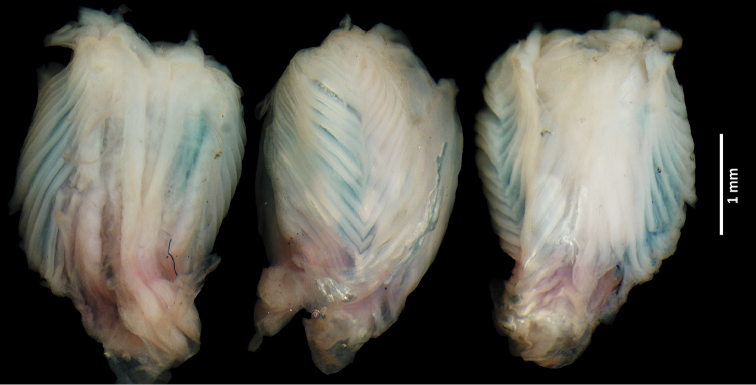
*Oreosaurus serranus* sp. n. Sulcate (left), lateral (center) and asulcate (right) views of the right hemipenis of ROM 53610 (paratype).

Right organ of subadult male ROM 53610 (Fig. 3) was partially everted and filled. Basal and lobular regions are partially damaged. Hemipenial body is roughly globose, ending in two small and partially everted, barely visible lobes. Partial eversion and some damages precluded the detection of folds, or any other ornamentation, on the lobes.

The sulcus spermaticus, central in position, originates at the base of the organ and proceeds in a straight line towards the lobes. It is bordered by two parallel nude areas, and divided by a fleshy fold. Branches of the sulcus spermaticus are not visible. Two columns of at least 14 chevron-shaped flounces ornament the sides of the organ and the borders of the sulcate and asulcate faces of the hemipenial body. Although these flounces do not present calcified comb-like spicules, it is possible that such absence is due to the age of the specimen. These calcified structures are present in adults of most species of Cercosaurinae that have their hemipenial morphology described, including species of *Oreosaurus* (e.g., Kok and Rivas 2011, Nunes 2011, Rivas et al. 2005). A broad nude area occupies at least 50% of the asulcate face. Some damages at the basis of the organ precluded the detection of the isolated horizontal flounces on the proximal-central region of the asulcate face that are often present in species of Cercosaurinae (e.g., Kok and Rivas 2011, Nunes 2011, Rivas et al. 2012, Sánchez-Pacheco et al. 2011).

#### Variation.

Paratypes consist of four females (SVL = 41.4–68.6 mm) and two males (SVL = 40.4–60 mm). The paratypes are similar to the holotype with the following noteworthy exceptions. Frontonasal longer than frontal in ROM 53609–12 and 53614; loreal scale in contact with second supralabial in ROM 53612–13; ventralmost postocular fused with posteriormost subocular on the right side in ROM 53613; medialmost scale of the posterior cloacal plate not divided horizontally in ROM 53610–11 and 53614; palpebral disc of the lower eyelid divided into two large, pigmented scales in ROM 53609; femoral pores per hind limb in female ROM 53612 three. Femoral pore number is the most evident sexually dimorphic character, with males having 7–9 pores per hind limb (ROM 53610 8/9, ROM 53614 9/7) and females having 2–3.

#### Distribution and natural history.

**Figure 4.**
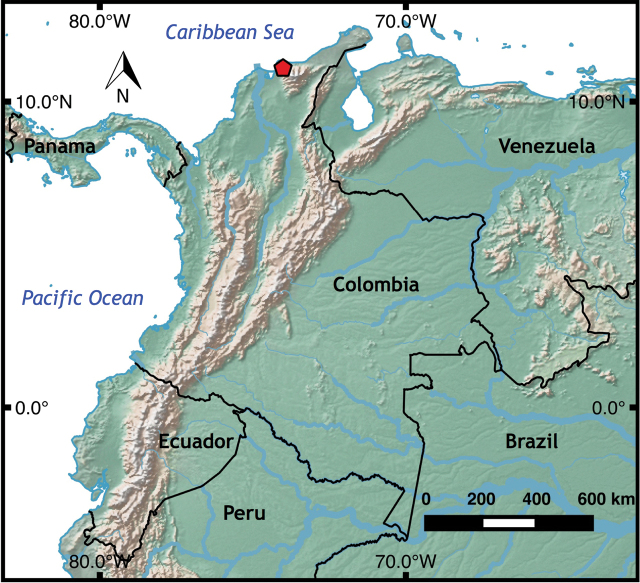
Distribution of *Oreosaurus serranus* sp. n. in the northwestern slopes of the Sierra Nevada de Santa Marta, northern Colombia. Pentagon indicates type locality.

**Figure 5.**
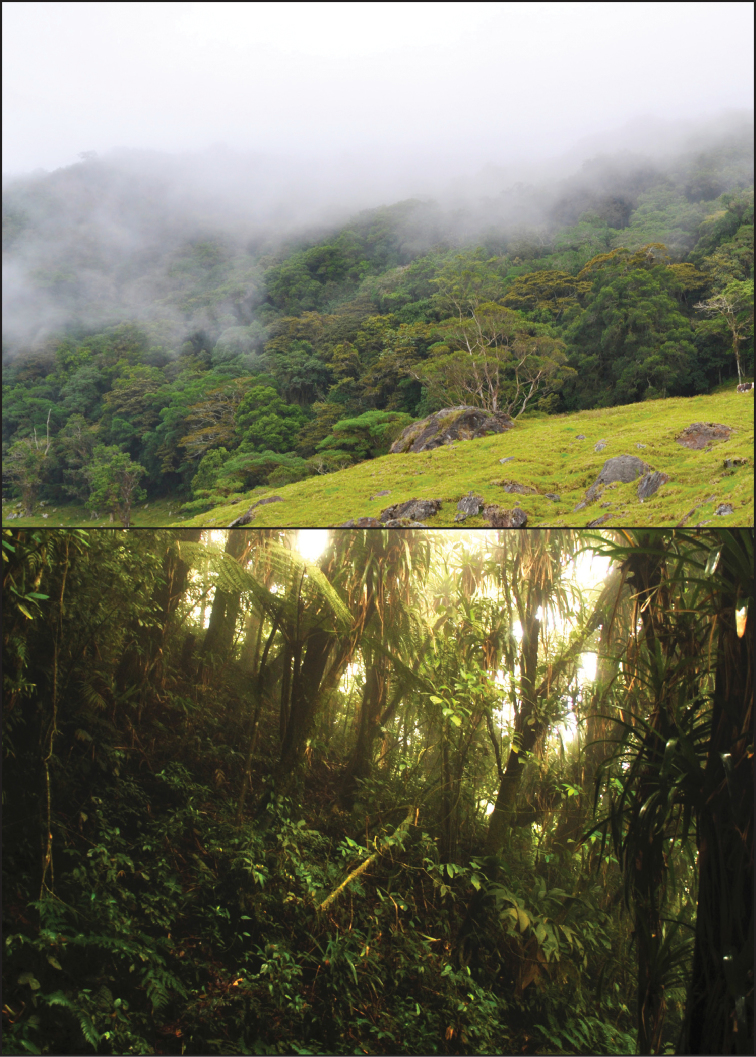
Type locality (top) and habitat (bottom) of *Oreosaurus serranus* sp. n. in the Sierra Nevada de Santa Marta, Colombia. Photos: Jhon Jairo Ospina-Sarria (top) and S.M.S (bottom).

*Oreosaurus serranus* sp. n. is known exclusively from the type locality (Figs 4, 5) and San Lorenzo (Ayala and Castro unpublished data, Ayala 1986), two adjacent cloud forest localities on the northwestern slopes of the Sierra Nevada de Santa Marta (SNSM) at elevations of about 1800–2156 m (Fig. 4). This forest-dwelling lizard is often found under fallen, rotten trunks or logs. Holotype and paratypes were collected manually during the day. The new species was found at the type locality in sympatry with *Anadia pulchella*, another gymnophthalmid endemic to the SNSM.

#### Etymology.

The specific epithet *serranus*, which is an adjective derived from the Spanish adjective serrano (meaning from the sierra), refers to the location of the species’ type locality in the Sierra Nevada de Santa Marta, and preserves the original etymological intent of Harris, as stated by Ayala and Castro (unpublished data).

#### Comments.

Formal nomenclatural recognition of *Oreosaurus serranus* sp. n. renders *specularis* (Ayala 1986) a permanently unavailable name for this taxon. Specimens reported by Ayala and Castro (unpublished data) were not included herein because they are presumably lost (S.J.S-P. personal observation).

*Oreosaurus* is one of the two genera extracted from the former *Riama* *sensu lato*, which was recently found to be non-monophyletic (Sánchez-Pacheco et al. 2017). The other clade, *Andinosaura* Sánchez-Pacheco et al., 2017, includes 11 Andean species and *Riama* *sensu stricto* is also an exclusively Andean radiation of 16 named species.

**Figure 6.**
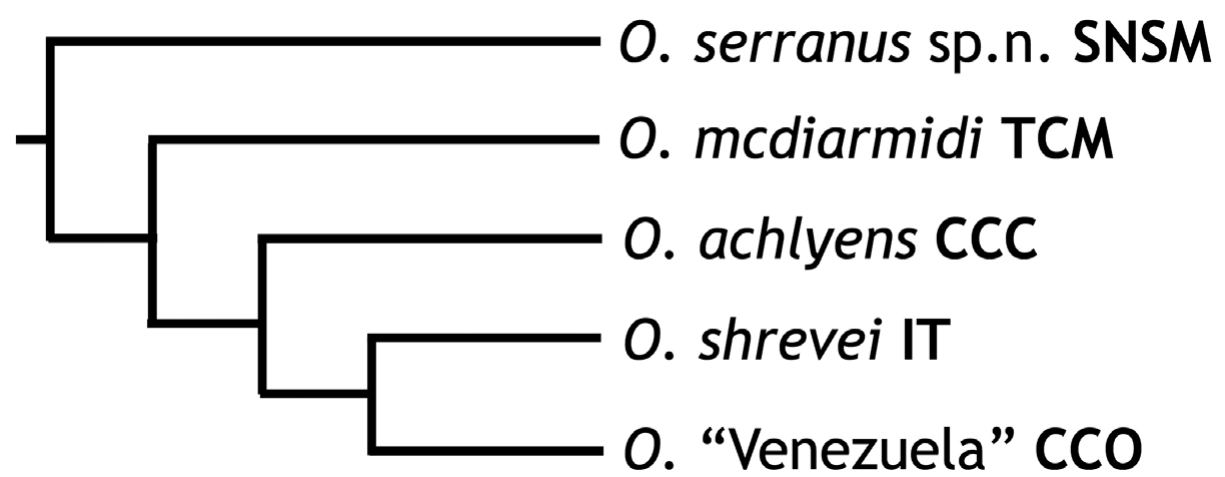
Summary of the phylogeny and geographic distribution of *Oreosaurus* (Sánchez-Pacheco et al. 2017). SNSM = Sierra Nevada de Santa Marta, Colombia; TCM = tepuis from the Chimantá massif, Venezuela; CCC = Cordillera de la Costa Central, Venezuela; IT = island of Trinidad; CCO = Cordillera de la Costa Oriental, Venezuela. *Oreosaurus luctuosus*, from the CCC, and *O. rhodogaster*, from the CCO, were included in this genus due to the presumed close relationships of these species and *O. achlyens* and *O. shrevei*, respectively. Data taken from Sánchez-Pacheco et al. (2017).

Sánchez-Pacheco et al. (2017) discussed the disjunct geographic distributions of species of *Oreosaurus*, as well as their phylogenetic relationships. Figure 6 summarizes these findings. All species of *Oreosaurus* share the absence of a narrow band of differentiated granular lateral scales (present in species of *Andinosaura* and *Riama*).

### Key to the species of *Oreosaurus*

**Table:** 

1	One pair of genial scales	***Oreosaurus serranus* sp. n.**
–	Two pairs of genial scales	**2**
2	Prefrontal scales present	***O. mcdiarmidi***
–	Prefrontal scales absent	**3**
3	Loreal scale absent	**4**
–	Loreal scale present	**5**
4	Anterior cloacal plate row composed of a small scale	***O. shrevei***
–	Anterior cloacal plate row composed of two large scales	***O.* “Venezuela**”
5	Dorsal body scales hexagonal	**6**
–	Dorsal body scales rectangular	***O. luctuosus***
6	42–44 transverse dorsal scale rows	***O. rhodogaster***
–	37–40 transverse dorsal scale rows	***O. achlyens***

## Supplementary Material

XML Treatment for Oreosaurus serranus

## Appendix 1

**Comparative material examined**

***Oreosaurus achlyens***: VENEZUELA: Aragua: Rancho Grande (AMNH 137260, 137267–69, 137271–76, 137278–82, 137297). ***O. luctuosus***: VENEZUELA: Aragua: Rancho Grande (AMNH 137270, 137277, MCZ 100410, USNM 196336), Parque Nacional Henry Pittier, Rancho Grande (USNM 259170). ***O. shrevei***: TRINIDAD & TOBAGO: Horne Tucuche (MCZ 62506–07); El Teluche [in error, probably Tucuche] (MCZ 100466–68); Mt. Tucuche (MCZ 160065–66). ***O.* “Venezuela**”: VENEZUELA: Anzoátegui: Cerro El Guamal, Macizo del Turimiquire, municipio Freites, 2150 m (EBRG 5962).
